# How previous treatment changes the metabolomic profile in patients with metastatic breast cancer

**DOI:** 10.1007/s00404-022-06558-5

**Published:** 2022-04-25

**Authors:** Juliane Nees, Simon Schafferer, Baowen Yuan, Quiqong Tang, Matthias Scheffler, Andreas Hartkopf, Michael Golatta, Andreas Schneeweiß, Barbara Burwinkel, Markus Wallwiener

**Affiliations:** 1 Department of Gynecology and Obstetrics, University of Heidelberg, Im Neuenheimer Feld 440, 69120 Heidelberg, Germany; 2 BIOCRATES Life Sciences AG, 6020 Innsbruck, Austria; 3 Molecular Biology of Breast Cancer, Department of Gynecology and Obstetrics, University of Heidelberg, 69120 Heidelberg, Germany; 4 Division of Molecular Epidemiology, German Cancer Research Center (DKFZ), 69120 Heidelberg, Germany; 5 Department of Women’s Health, University of Tübingen, 72076 Tübingen, Germany; 6 National Center for Tumor Diseases (NCT), 69120 Heidelberg, Germany

**Keywords:** Metastatic breast cancer, Metabolomics, Serine, Methionine, Circulating tumor cells

## Abstract

**Purpose:**

Metabolites are in the spotlight of attention as promising novel breast cancer biomarkers. However, no study has been conducted concerning changes in the metabolomics profile of metastatic breast cancer patients according to previous therapy.

**Methods:**

We performed a retrospective, single-center, nonrandomized, partially blinded, treatment-based study. Metastatic breast cancer (MBC) patients were enrolled between 03/2010 and 09/2016 at the beginning of a new systemic therapy. The endogenous metabolites in the plasma samples were analyzed using the AbsoluteIDQ^®^ p180 Kit (Biocrates Life Sciences AG, Innsbruck) a targeted, quality and quantitative-controlled metabolomics approach. The statistical analysis was performed using R package, version 3.3.1. ANOVA was used to statistically assess age differences within groups. Furthermore, we analyzed the CTC status of the patients using the CellSearch^™^ assay.

**Results:**

We included 178 patients in our study. Upon dividing the study population according to therapy before study inclusion, we found the following: 4 patients had received no therapy, 165 chemotherapy, and 135 anti-hormonal therapy, 30 with anti-Her2 therapy and 38 had received treatment with bevacizumab. Two metabolites were found to be significantly different, depending on the further therapy of the patients: methionine and serine. Whereas methionine levels were higher in the blood of patients who received an anti-Her2-therapy, serine was lower in patients with endocrine therapy only.

**Conclusion:**

We identified two metabolites for which concentrations differed significantly depending on previous therapies, which could help to choose the next therapy in patients who have already received numerous different treatments.

## Introduction

### Breast cancer

Breast cancer (BC) is the most common female cancer all over the world. Whereas over 90% of patients diagnosed with primary cancer survive the first 5 years, only 15% of patients with advanced BC live more than 5 years [1]. These facts underline the significance of improving therapy monitoring, especially in patients with metastatic BC (MBC), to avoid ineffective therapies. Metastatic patients usually receive numerous different kinds of therapies so that sometimes it is difficult to find a new treatment when disease is progressing. With all the new therapeutic options that are available, a physician is left alone with non-tested and non-approved combinations to choose the next therapeutic regimen [2]. The established methods for monitoring therapy include procedural imaging [e.g., magnetic resonance imaging (MRI) and computed tomography (ct)], and blood tests, such as CA15-3 and CEA. Blood tests seem to be very attractive but they are not sensitive and specific enough [3]. Among the new methods for monitoring therapy and predicting overall and progression-free survival, is measuring the circulating tumor cells (CTC) providing deeper prognostic information than state-of-the-art imaging [4] and may help to identify patients with a high therapy pressure due to bad prognosis without an aggressive therapy [5]. Nevertheless, CTC are not used in the clinical routine until today. All in all, new and better markers are needed for predicting therapy response and therapeutic monitoring.

### Metabolomics

Metabolomics, measuring low molecular weight molecules, represents a recent development attempting to comprehensively and quantitatively, representing a lot of intermediary metabolism pathways by covering systematically the key metabolites [6]. Metabolomics depict functional information and bridge this information gap [7]. Differences in metabolites found in fluid or tissues provide the closest connection to the various phenotypic responses; changes might both provide new diagnostic information and help develop therapeutic options for malignant diseases. Metabolites can be detected in the tissue, blood, urine and salvia of patients and are therefore very easy to retrieve. Recent analysis of the function of tumor suppressors and oncogenes discovered that many of them play an important role in cell metabolism [8]. In particular, changes in the metabolic systems of cancer cells in comparison to healthy include the induction of the cell membrane and the use of glucose through non-oxidative pathways [9]. In patients with prostate cancer, androgen deprivation therapy changed the metabolomics profile: steroid levels dropped, bile acids increased, and biomarkers of lipid metabolism and insulin resistance decreased [10]. In BC, significant chances in the metabolomics profile could be also detected between healthy controls and early stage cancer patients [11].

### Methionine

Methionine is an essential amino acid and cannot be synthesized by humans. Its three main functions are glutathione formation via cysteine, methyl group donation for example at DNA modification via methylation and polyamine synthesis [12] which always starts with methionine on the N-terminus. Methionine can be converted to S-adenosylmethionine (SAM) by linking with ATP through methionine adenosyltransferase, an important methyl group donor in most organisms. After giving the methyl group, S-adenosyl homocysteine (SAH) is formed, which is ultimately converted to homocysteine and from which methionine is recovered. Tissues with a high turnover rate require more methionine to produce proteins they need to grow. Furthermore, methionine being leading targets is converted to methionine sulfoxideby the influence of reactive oxygen species [13].

### Serine

Serine is a proteinogenic nonessential amino acid playing an important role in activating and inactivating several enzymes, especially peptidases. Serine can either be synthesized from glucose as a component of the glycolysis-diverting pathway [14] or imported from the extracellular environment. In detail, glucose-6-phosphate is formed to 3-phosphoglycerate and via phosphoglycerate dehydrogenase into the serine precursor 3-phosphohydroxypyruvate. 3-Phosphohydroxypyruvate is converted into serine via transamination and phosphate ester hydrolysis reactions. This biosynthesis is said to play an important role for many cancers due to its link to nucleotide biosynthesis [15]. And its pathway with one-carbon is upregulated metastatic sub-clones of the BC cell line MDA-MB-231 driving faster proliferation [16].

### Circulating tumor cells

Using the CellSearch^™^ system (Veridex), CTCs can be detected in about 50% of patients with MBC [17]. A positive CTC status (≥ 5 CTCs/7.5 ml peripheral blood) correlates with shorter PFS and OS [18–20]. Furthermore, serial CTC enumeration is an effective test for predicting treatment outcome in MBC and a versatile addendum to classic diagnostic tools for therapy tailoring [21].

### Metabolomics in BC

There is explicit evidence that currently established clinical diagnostic tools for BC can be complemented by metabolomics to, including the possibility to detect BC in early stages [22], relapse [23] and to predict treatment response, OS and PFS by analyzing blood and tissue samples.

To identify new therapeutic targets, deeper knowledge of the molecular changes depending on subtypes is essential to monitor treatment responses [24]. Recent studies found the estrogen receptor (ER) status of the tumor influencing the glutamate-to-glutamine ratio (GGR). That ratio can also be used to predict PFS and OS: a higher level is associated with longer OS [25]. Budczies et al. also found that the GGR significantly correlated with tumor grade and the ER status.

Triple-negative breast cancer (TNBC) is reported as a very metabolic disease [26] with elevated energy metabolism pathways that may directly impact the aggressive nature of this cancer [27].

### Objective

The objective of this study was to gain a better understanding of the changes in metabolism in MBC according to the previous treatment. Furthermore, the CTC status of the patients was analyzed at study inclusion.

## Methods

### Patients

MBC patients were consecutively included in the study between 2010 (March) and 2016 (September) at the start of a new line of systemic therapy selected by the attending physician independent from the current study. Their blood was analyzed to determine the metabolomics profile. Previous treatments were clustered into chemotherapy, endocrinological therapy (ET), targeted therapy against human epidermal growth factor receptor 2 (Her2), and therapy with bevacizumab and the combination of these different therapy strategies. Subsequently, the metabolomics profile according to the previous treatment regimen was compared.

### Blood sample processing

Blood samples (30 ml) were collected in standard 10-ml tubes containing ethylene-diamine-tetra-acetic acid and a cellular preservative before patients received surgery or chemotherapy. Before being analyzed, the samples were centrifuged 20 min at 2700 rpm (1300×*g*) at 10 °C. The blood pellets were put in small cryo tubes (1 ml) and shock frozen in liquid nitrogen in a −80 °C freezer. The plasma was transferred into 2 ml tubes and again centrifuged 10 min at 1200 rpm (12,000×*g*) and the supernatant was put in small cyro tubes and shock frozen.

### Targeted metabolome analysis using LC–MS/MS

The plasma with the endogenous metabolites was analyzed with a targeted quality, and quantitative-controlled metabolomics approach using the AbsoluteIDQ^®^ p180 Kit (Biocrates Life Sciences AG, Innsbruck), as published previously [28, 29]. With this kit up to 188 endogenous metabolites can be quantified, including 21 biogenic amines, 21 amino acids, 90 glycerophospholipids, 40 acylcarnitines, 15 sphingolipids, and hexoses. Liquid chromatography tandem mass spectrometry (LC–MS/MS) was applied to detect amino acids and biogenic amines, whereas using flow injection analysis tandem mass spectrometry (FIA-MS/MS) we could quantify acylcarnitines, sphingolipids, glycerophospholipids, and hexoses. Sample preparation was performed according to the user manual of the kit. Samples were randomized, including multiple quality control samples in the measurement sequence. The complete analytical process (sample registration, work list generation, and data processing) was carried out using the Biocrates MetIDQ^™^ software, version Boron 2693 (Biocrates Life Sciences AG, Innsbruck), which is an integral part of the AbsoluteIDQ^®^ p180 Kit. Metabolite concentrations were calculated using the MetIDQ^™^ software and reported in μmol/l.

### CTC detection

7.5 ml peripheral whole blood was drawn in a CellSave tube (J Janssen Diagnostics, LLC, Raritan, NJ, USA) for CTC enumeration. Blood samples were kept at room temperature for  ≤ 96 h until analysis using the CellSearch^™^ assay (CellSearch^™^ Epithelial Cell Kit/CellSpotter^™^ Analyzer, Janssen Diagnostics, LLC, Raritan, NJ, USA). Sample analysis and processing were done exactly according to the manufacturer’s instructions [5].

### Study design

The study was performed as a retrospective, single-center, nonrandomized, treatment-based, partially blinded study. Treating physicians and patients were blinded to metabolomics status. The selection of the next system therapy was not based on the metabolic profile. All technical staff and investigators performing or reviewing the metabolomics were blinded to patient (treatment) history. The study was conducted at the National Center for Tumor Diseases (NCT), Heidelberg, Germany, and the Department of Obstetrics and Gynecology, University of Heidelberg, Heidelberg, Germany.

### Statistical analysis

An analysis of variance (ANOVA) was done to statistically assess age differences within groups. To statistically assess the differences in metabolite concentrations between CTC groups and healthy controls, a *t *test was conducted with equal variances to consider different sample sizes. Since comparisons for each metabolite were made, a multiple testing correction procedure by Benjamini and Hochberg was done to calculate the false discovery rate (FDR) as presented in Tables 3 and 4. To consider significant differences of contributing factors between groups, Tukey’s range test (Tukey 1949), Kruskal–Wallis rank-sum test, and the Pearson’s Chi-squared test were executed and results are presented in Table 1. A *p *value (FDR) of ≤ 0.05 was regarded as significantly different (*).

**Table 1:** Patient characteristics

Parameter	Statistics	Anti-Her2 group	Exclusively ET Group	Study population	*p *value
Age	Median	53	65.5	59	*1.63E−02
	Range	36–72	52–78	31–89	
Bone metastasis					
No	*N* (%)	18 (60)	6 (75)	89 (50)	1.41E−01
Yes	*N* (%)	12 (40)	2 (25)	89 (50)	
Liver metastasis					
No	*N* (%)	21 (70)	7 (87.5)	119 (66.9)	3.89E−01
Yes	*N* (%)	9 (30)	1 (12.5)	59 (33.1)	
Lung metastasis					
No	*N* (%)	19 (63.3)	5 (62.5)	116 (65.2)	9.57E−01
Yes	*N* (%)	11 (36.7)	3 (37.5)	62 (34.8)	
T					
T1	*N* (%)	10 (33.3)	2 (25)	54 (30.3)	8.83E−01
T2	*N* (%)	13 (43.3)	3 (37.5)	82 (46.1)	
T3	*N* (%)	3 (10)	0 (0)	16 (9)	
T4	*N* (%)	3 (10)	2 (25)	19 (10.7)	
Tis	*N* (%)	0 (0)	0 (0)	1 (0.6)	
NA	*N* (%)	1 (3.3)	1 (12.5)	6 (3.4)	
Grading					
G1	*N* (%)	1 (3.3)	1 (12.5)	7 (3.9)	1.84E−01
G2	*N* (%)	10 (33.3)	6 (75)	82 (46.1)	
G3	*N* (%)	13 (43.3)	0 (0)	66 (37.1)	
NA	*N* (%)	6 (20)	1 (12.5)	23 (12.9)	
Estrogen receptor (ER)					
Positive	*N* (%)	15 (50)	8 (100)	134 (75.3)	*8.37E−04
Negative	*N* (%)	15 (50)	0 (0)	39 (21.9)	
NA	*N* (%)	0 (0)	0 (0)	5 (2.8)	
Progesterone receptor (PR)					
Positive	*N* (%)	13 (43.3)	6 (75)	116 (65.2)	*1.08E−02
Negative	*N* (%)	17 (56.7)	2 (25)	54 (30.3)	
NA	*N* (%)	0 (0)	0 (0)	8 (4.5)	
Her2					
Negative	*N* (%)	8 (26.7)	8 (100)	133 (74.7)	*2.63E−16
Positive	*N* (%)	19 (63.3)	0 (0)	24 (13.5)	
NA	*N* (%)	3 (10)	0 (0)	21 (11.8)	
CTC					
Negative	*N* (%)	25 (83.3)	5 (62.5)	110 (61.8)	*1.40E−05
Positive	*N* (%)	5 (16.7)	1 (12.5)	64 (36)	
NA	*N* (%)	0 (0)	2 (25)	4 (2.2)	
Surgery					
No	*N* (%)	7 (23.3)	3 (37.5)	28 (15.7)	8.05E−02
Yes	*N* (%)	23 (76.7)	5 (62.5)	150 (84.3)	
Radiation					
No	*N* (%)	16 (53.3)	6 (75)	67 (37.6)	*7.80E−03
Yes	*N* (%)	14 (46.7)	2 (25)	111 (62.4)	

Anti-Her2 Group are the patients who received anti-Her2 therapy before being included in the study: exclusively ET group are the patients who did not receive any other therapy except for endocrine therapy

## Results

### Patient clinical characteristics

178 patients with metastatic breast cancer in were included in this study (Table 1). The median age was 59 years and ranged from 31 to 89 years. Half of the study population suffered from bone metastasis, 33% had liver metastasis, and 35% metastasis of the lung. When first diagnosed with breast cancer, 30% had a T1 tumor stage, 46% a T2 stage, and 9% T3 stage. One patient only had ductal carcinoma in situ (DCIS) and with unknown initial tumor stage of 3% of the patients. Most of the tumors were intermediate grade (46%), 37% were high-grade, and 4% low-grade tumors. The tumor grade is unknown for 13%. Most of the patients (75%) had an ER-positive tumor, 22 were ER negative, and 3% are unknown. Concerning the PR status 65% were positive, 30% were negative, and of 5% indefinite. Most of the patients had a Her2 negative tumor (75%); only 14% were Her2 positive, and 12% are unknown. Of the patients 62% were CTC negative and 36% positive; for 2% of the patients, the CTC status was unknown. Most of the patients (84%) had received surgery before being included in the study and 62% received radiation.

When the study population was divided according to therapy before study inclusion (see Table 2), it was found that 4 patients had received no therapy before being included in the study, 165 had received any chemotherapy, and 13 no chemotherapy at all. Seventeen patients had chemotherapy only and no other therapy. In all, 135 patients had received any kind of anti-hormonal therapy, and 43 did not have such therapy. Eight of our patients had systemetically anti-hormonal therapy only. In all, 30 MBC patients were treated with anti-Her2 therapy, and 148 were not. Finally, 38 patients in our study received therapy with bevacizumab, and 140 did not have such a therapy.

**Table 2:** Patient numbers according to the previous therapy

Therapy	Yes	No	Exclusively
Any	174 (97.8%)	4 (2.2%)	–
Chemotherapy	165 (92.7%)	13 (7.3%)	17 (9.5%)
Endocrine therapy	135 (75.8%)	43 (24.2%)	8 (4.5%)
Anti-Her 2	30 (16.9%)	148 (83.1%)	0
Bevacizumab	38 (21.3%)	140 (78.7%)	0

### Differences in the concentrations of metabolites

**Fig. 1:**
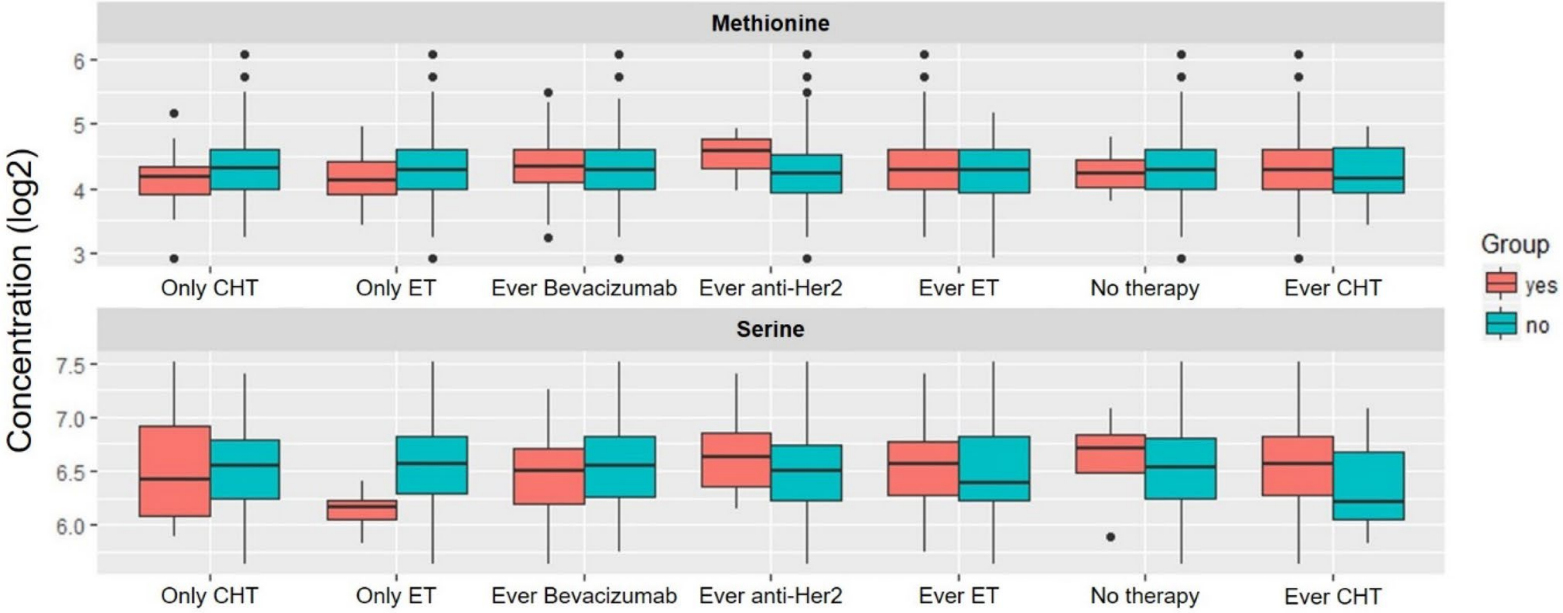
Plasma levels of methionine (upper panel) and serine (lower panel) to different previous therapies. *CHT* chemotherapy, *ET *endocrine therapy

There is a significant difference in the metabolomics profile between patients who received anti-Her2 therapy and those who did not receive such a therapy. The patients who received therapy had higher concentrations of methionine in their blood than the patients who did not receive anti-Her2 therapy (see Table 3). We also found a significant difference in the patients who were treated with endocrine therapy only: concentrations of serine were lower than in the other patients (see Table 4).The concentration differences depending on the previous therapy for serine and methionine are shown in Fig. 1.

**Table 3:** Metabolomics according to therapy against Anti-Her2

Metabolite	pVal	FDR	Fold Change	log2FC
Met	0.000225	*0.033471	1.14504	0.195398
Met-SO	0.00418	0.311396	1.183643	0.243233
C5	0.029313	0.796629	−1.71614	−0.77917
GDCA	0.053344	0.796629	1.613991	0.690632
SM C18:0	0.057353	0.796629	−1.10374	−0.1424
Ser	0.061827	0.796629	1.102561	0.140859
PC aa C42:0	0.0629	0.796629	1.128321	0.174178
Tyr	0.068192	0.796629	1.069945	0.097537
His	0.068498	0.796629	1.047076	0.066366
Gln	0.075358	0.796629	1.056773	0.079666

*FDR ≤ 0.05 (regarded as significantly different)

*pVal*  *p* value; *FDR*   false discovery rate; *Log2FC*   log2 fold change; *Met *methionine; *Met-SO*   methionine sulfoxide; *C5*  valerylcarnitine; *GDCA*  glycodeoxycholic acid; *SM C18.0* sphingolipid octadecanoylcarnitine, *Ser* serine; *PC aa C42.0* glycerolipid dotetracontaylcarnitine *Tyr* tyrosine; *His* histidine; *Gln* glutamine

**Table 4:** Differences in the metabolomics for patients with only endocrine therapy

Metabolite	pVal	FDR	Fold change	log2FC
Ser	9.92E-05	*0.014775	−1.37295755	−0.45729
TCDCA	0.002563	0.129588	−8.16052906	−3.02866
TUDCA	0.002609	0.129588	−4.17712275	−2.06251
Spermine	0.005563	0.207221	1.16611114	0.221705
TCA	0.039891	0.984027	−20.4249691	−4.35226
PC ae C36:4	0.058837	0.984027	1.21862174	0.28525
PC aa C28:1	0.062185	0.984027	1.11377595	0.155459
SM C18:0	0.12694	0.984027	1.22327802	0.290752
lysoPC a C16:1	0.133064	0.984027	−1.31319242	−0.39308
PC ae C38:5	0.133169	0.984027	1.19877895	0.261566

* FDR ≤ 0.05 (regarded as significantly different)

*pVal* *p* value; *FDR* false discovery rate; *Log2FC* log2 fold change; *Ser* serine; *TCDCA* taurochenodeoxycholic acid; *TUDCA* tauroursodeoxycholic acid; *TCA* taurocholic acid; *PC ae C36.6* glycerolipid hexatriacontylcarnitine; *PC ae 28.1* glycerolipid octacosaylcarnitine; *SM C18.0* sphningolipid octadecanoylcarnitine, *lysoPC a C16:1* lysophposphatiidaycoline hexadecenoylcarnitine; *PC ae C38:5* glycerolipid octatriacontylcarnitine

Neither other significant changes were found in the metabolomics profile for the patients who received chemotherapy or bevacizumab, nor were any significant differences discovered in the combination of two or three therapeutic options.

## Discussion

It is already known that the metabolomics levels depend on internal factors, such as gender, age, race and (cancerous) disease, but also on externals for example nutrition and therapies, e.g., medicinal but also radiation [30, 31]. In breast cancer patients, metabolomics concentrations are additionally dependent on tumor type (ER, PR, and Her2) [32] and the extent and site of metastasis [33, 34].

### Methionine

A statistically significant difference was found in the concentration of methionine, which plays a rate-limiting role in tumorigenesis [35], associated with anti-Her2 therapy. The patients who had received this treatment had a higher concentration of methionine in their blood than the patients who did not receive anti-Her2 therapy. Willmann et al. previously reported changes in the methionine cycle at breast cancer cell lines. There significantly different levels of SAM, SAH, MTA (5 deoxy 5 methylthioadenosine), MTA-SO (sulfoxide), and 3-(3-Amino-carboxypropyl)-uridine were identified in breast cancer cells compared to normal breast tissue [36]. An elevated level of SAM was detected in the Her2-positive cell line. Thus, the elevated methionine levels found might have been caused by the Her2-positive tumor and not the therapy. SAH was elevated in TNBC and hormone receptor-negative and Her2-positive breast cancer but not in hormone receptor-positive and Her2-positive breast cancer. MTA and MTA-SO levels were lower in all BC cell lines, probably because they were involved in the recycling process of methionine.

### Serine

Significantly lower concentrations of serine were observed in patients who received endocrine therapy only. Similar data were published by Kim et al., who reported higher serine levels for patients with TNBC but low serine concentrations in the blood of patients with luminal A cancer [37] who normally are treated with endocrine therapy only. Related to pre-therapies, serine accumulation has been described at PARP inhibition [38]. Furthermore, Possemato et al. found increased serine synthesis flux for patients with estrogen receptor-negative breast cancer, which is also associated with poor 5-year survival [39]. Thus, changes in metabolite concentrations can also be useful for predicting overall survival for breast cancer patients. Additionally, changes in serine concentration may be caused by different tumor types, which are treated differently, and therefore changes in the metabolite concentrations may be related to the tumor type and not the therapies.

As for challenges in interpreting these results, some subgroups are small and the heterogeneity of the study population and their primary tumor is high, which was not correlated in the description of the metabolomics profile.

## Conclusion

There are significant differences in the metabolomics profile of MBC patients according to their previous therapy. Patients who received anti-Her2 therapy had a higher concentration of methionine in their blood than the patients who did not have such a therapy. Furthermore, serine concentrations were lower in patients who were treated with endocrine therapy only. These findings and further metabolomics studies may help to find new therapeutic strategies for the individual patient and perhaps to monitor treatment by determining changes of metabolites during therapy.

## Outlook

In future, metabolomics might help to improve diagnosis, therapeutic monitoring, and aid in finding new therapy targets and drugs such as AG-221, an inhibitor of mutated iso-citrate dehydrogenase 2 (IDH2) for patients with acute myeloid leukemia [26].

Metabolomics offer the possibility to improve diagnosis, disease control, and monitoring breast cancer therapy with high sensitivity and specificity in a low invasive way [3]. They may help to detect relapse and progression and to discover breast cancer early in patients when other methods are not sensitive enough. Metabolomics may also support to improve treatment strategies [40].

## Data Availability

The authors confirm that the data supporting the findings of this study are available within the article.

## Abbreviations

ANOVA: Analysis of variance
CA15-3: Cancer-antigen 15-3
CEA: Carcinoembryonic antigen
CT: Computed tomography
CTC: Circulating tumor cells
DCIS: Ductal carcinoma in situ
EDTA: Ethylenediaminetetraacetic acid
ER: Estrogen receptor
ET: Endocrinological therapy
FDR: False discovery rate
FIA-MS/MS: Flow injection analysis tandem mass spectrometry
GGR: Glutamate-to-glutamine ratio
Her 2: Human epidermal growth factor receptor 2
IDH 2: Isocitrate dehydrogenase 2
LC–MS/MS: Liquid chromatography tandem mass spectrometry
MBC: Metastatic breast cancer
MTA: 5 Deoxy 5methylthioadenosin
NCT: National Center for Tumor Diseases
PR: Progesterone receptor
SAH: S-Adenosyl homocysteine
SAM: S-Adenosylmethionine
TNBC: Triple-negative breast cancer

## Code availability

R software, version 3.3.1.
